# Does Bridge Employment Mitigate or Exacerbate Inequalities Later in Life?

**DOI:** 10.1093/geroni/igab046.1603

**Published:** 2021-12-17

**Authors:** Kevin Cahill, Michael Giandrea, Joseph Quinn, Lawrence Sacco, Loretta Platts

**Affiliations:** 1 Center on Aging & Work at Boston College, Chestnut Hill, Massachusetts, United States; 2 U.S. Bureau of Labor Statistics, Washington, District of Columbia, United States; 3 Boston College, Chestnut Hill, Massachusetts, United States; 4 Stockholm University, Stockholm, Stockholms Lan, Sweden; 5 Stress Research Institute, Department of Psychology, Stockholm University, Stockholm, Stockholms Lan, Sweden

## Abstract

This paper explores how gradual retirement impacts inequality later in life, with a focus on transitions from career to bridge employment. We use 26 years of longitudinal data from the Health and Retirement Study to document the various pathways that older Americans take when exiting the labor force, and examine how bridge employment impacts non-housing wealth and total wealth, including the present discounted value of Social Security benefits. We find that gradual retirement in the form of bridge employment neither exacerbates nor mitigates wealth inequalities among Americans who held career jobs later in life. We do find evidence that wealth inequalities grow among the subset of older career workers who transition from career employment to bridge employer at older ages. These findings provide quantitative evidence that bridge employment at older ages is taken by those who need to continue working financially and those who continue working for nonpecuniary reasons.

